# Hospital Meetings

**Published:** 1906-03-03

**Authors:** 


### HOSPITAL MEETINGS.

### EAST LONDON HOSPITAL FOR CHILDREN.

This is one of the best managed of children's hospitals anywhere in the world. One striking test of the quality of the management of a children's hospital is the quietness of the wards. In making our inspection we find children's hospitals where children will be found crying in several of the wards. A little close investigation usually reveals the cause, for children never cry unless they are in pain or discomfort, and the test of a sister and nurse's capacity to have charge of a children's ward is hence the absence of noise. The East London Hospital is a striking proof of the justice of this criticism, for it is probably the quietest of children's hospitals. Any rich persons suffering from ennui and disgust on a dull, foggy, miserable day in London, should make it their business to go to Shadwell and visit this hospital. The contrast between the outside and inside atmosphere on such a day will be an experience to make them pause and think. This is another severe test of the quality of the management which we have always recognised with pleasure.

The Earl of Erroll presided at the thirty-seventh annual meeting of the East London Hospital for Children and Dispensary for Women, Shadwell, on the 26th ultimo. In opening the proceedings he expressed regret that the hospital was not as well known as many. They were greatly handicapped, he said, by their situation. Few people in the West End even knew of their existence, or indeed where Shadwell was. It was not right that such an institution should have to come cap in hand to the public for funds to carry on their work, and he could not understand how that public, which benefited by the experience gained in hospitals, could reconcile it with their consciences not to support them better than they did. He should also like to call special attention to the Convalescent Home at Bognor, since many of the cures effected would be incomplete and of little value unless they were able to send the patient to the seaside.

Colonel Charles Needham, Chairman of the Board of Management, proposed the adoption of the report and accounts. Going briefly through the report, he pointed out that the number of in-patients for the year showed a decrease of 69—1,587 against 1,656—and the daily average of patients was less by 2.29. The cost of maintenance per patient per week had increased from £1 2s. 2d. to £1 5s. The reason for the decrease in numbers was that, owing to outbreaks in the wards, they had had to be closed for disinfection, and also to the longer period taken for the annual cleaning, which was being done more thoroughly than in former years. The increased cost was also due to the opening of 12 extra beds in the new whooping-cough block. The number of out-patients had decreased from 31,312 to 29,855, and this was due to more discrimination on the part of the medical officers. Without an almoner, however, they could not hope altogether to eliminate those who ought not to come upon a hospital for relief, but in the present state of their finances the Board did not see their way to the additional expense this would involve. Looking at the accounts it would be seen that there was an increase of £674 in ordinary receipts, chiefly due to a rise in donations, in the grants from the Sunday and Saturday Funds, and in the receipts from the festival dinner of £250. On the other hand, subscriptions had dropped by £56. Legacies showed an increase of £2,759. The total maintenance expenditure showed a heavy increase of £390, due to repairs, extra cleaning, and the new whooping-cough block. Within the past two years they had accomplished important work. Two new blocks—one an isolation block—had been designed, completed, and paid for without trenching on the funds of the hospital, and this work had already abundantly proved its utility. But he must emphasise the fact that though the apparent deficit at the end of the year of £75 seemed small, it was really misleading, because the whole of the legacies had been spent, and only £1,000 had been reinvested of the

£2,000 sold out at the beginning of the year. They were still in very urgent need of funds, and he specially wished to draw attention to the forthcoming festival dinner on June 5th, when Lord Brassey would occupy the chair. He might mention that the Board had under consideration the question of making the hospital better known by means of advertisements.

Mr. Prescott, the Treasurer, seconded the resolution, which was carried. The general committee was reappointed, and some other formal business brought the proceedings to a close.

### ROYAL LONDON OPHTHALMIC HOSPITAL.

This hospital is making excellent progress, and its hold upon the confidence of the public is eloquently testified by the fact that the annual subscriptions have increased, and that the sum received from legacies exceeded £21,000. We feel that the speakers missed a great opportunity in not dwelling upon the importance of the educational work being carried on within the walls of this institution. A point should have been made, too, of the steady and successful effort which has been made to establish a sinking fund to wipe out a heavy charge represented by the large ground-rent which has to be met owing to the selection of an expensive site, with the object of securing a central position readily accessible from all parts of the metropolis. The record of this hospital during decades of years is one which ought always to be emphasised, in our judgment, at the annual meeting, because it is very remarkable, and proves to demonstration the great value of this, the largest and greatest of our ophthalmic hospitals, to the public and to the medical profession.

Lord Avebury presided at the annual meeting of the Royal London Ophthalmic (Moorfields Eye) Hospital on Wednesday, February 22. In moving the adoption of the report and accounts for 1905 he said that although there was nothing very thrilling to record, their affairs were always interesting. The amount of good they had been enabled to do was as much as that in the previous year, and a considerable advance on those before it, but it could not be said that their finances were by any means all they could wish. Their subscriptions had risen from £1,710 to £1,805; but their donations had fallen off from £2,503 to £1,535, and the committee had had to use over £2,000 from legacies for current expenses. They were thankful to the three great Funds for their contributions. From the Hospital Sunday Fund they had received £1,282; from the Saturday Fund £272, and from King Edward's Fund not only their annual grant of £2,000, but also a special donation of £1,500, and the Distribution Committee had placed it on record that in their opinion the hospital should receive more support from the public. As to expenditure, it was not desirable that they should attempt to make any great reduction. He was glad to point out that they had received during the year three legacies of £1,000, and one, from Mr. Herbert Cove, of £18,000. He should like to take the oportunity of impressing on them that they had now a fine building, an efficient Committee, and a most admirable staff, and they ought to be able to utilise them to the fullest extent. Their in-patients during the year had numbered over 2,000; and the attendances of out-patients totalled nearly 128,000. They must remember, too, that they had a great school, where most important improvements and discoveries were made in ophthalmic science which were of the greatest good to the whole world in relieving suffering from the eye. They hoped next year for such an improvement in their financial position as would enable them to open the twenty beds still closed, and so be able to give all the assistance possible.

Mr. Sturgis seconded the motion, which was agreed to, and Mr. Davison, in replying to a vote of thanks to the Committee, made a special appeal to the members of the Labour Party in the new House of Commons to free the hospitals of London from the incubus of rates which oppressed them, and so enable them to do more for the suffering poor.

### ROYAL FREE HOSPITAL.

Mr. Charles Burt, the treasurer, in his speech moving the adoption of the report, was in our view unduly pessimistic, seeing that, although the total expenditure (£14,093) was some £600 more than the total income (£13,413), the ordinary income of 1905 was, in fact, upwards of £1,300 more than that of the previous year. The whole deficiency was caused by a falling-off in the extraordinary revenue which in the previous year included £1,000 for the endowment of a bed, and the receipt of £3,300 more from legacies in 1904 than in 1905. Despite this fact the committee were, however, actually in funds, owing to the circumstance that £1,000 was received from the Zunz Fund, which enabled them to more than meet all their liabilities. We have held for years that the Royal Free Hospital is an institution which must command the goodwill and support of all thinking and intelligent supporters of hospitals, because of its excellent financial arrangements. The figures for the two last years confirm this view, and, instead of being pessimistic, in our view the Treasurer had good cause to be well satisfied and to speak with great confidence of the future by the experience of the past. Another fact in regard to this meeting which calls for comment is the circumstance that not a word was said about one of the most excellent features of the year's work and the system of management pursued at the Royal Free Hospital. For some years it has been the practice to take a census of all the patients in the wards on the second Sunday in January. This plan should be followed by every metropolitan hospital, because it enables the managers to show what proportion of the in-patients are resident in the district served by the hospital, the actual number of patients residing in other London districts, and also the numbers which have been sent in from the country. The census taken on the second Sunday in January 1906 is interesting from the circumstance that it shows that at that date there was a relatively small number of residents in the immediate district served by the hospital, whilst the numbers received from other London districts and from the country were greater than those in any one of the previous four years. It is true that the patients who reside in the immediate neighbourhood are as 87 to 64, a fact which testifies to the popularity of the hospital with the people who have every opportunity of knowing most about the efficiency of the work done, and this should encourage many to give liberally to its support.

There was a good attendance at the seventy-eighth annual meeting of the Royal Free Hospital, which was held on February 21.

The chair was taken by the Earl of Sandwich.

The adoption of the report was moved by Mr. Charles

Burt, Treasurer, who said the hospital had now attained quite a respectable age, being seventy-eight years old, and he hoped it would go on and prosper for many more years. The most important item in the report was the account of the structural improvements which had taken place in the hospital. Most of the chief London hospitals had been engaged in the same task of late years. It was very satisfactory that they had been able to pay for all the work which had been done, and they felt that it was money well laid out. At no time in the history of the hospital had it been in such an excellent condition as it was now. During the past year 2,329 in-patients had been treated, and they had each stayed on an average eighteen or nineteen days. The number of out-patients and casualties amounted to 42,720, while 431 maternity cases had been treated. This department had only been taken up during the last year or two, and it had already been of great service to the poor women of the neighbourhood. Their income for 1905 amounted to £13,413, including very handsome contributions from King Edward's Hospital Fund, the Hospital Sunday Fund and Hospital Saturday Fund. The total was, however, less by £3,000 than that received in 1904, chiefly owing to a falling-off in legacies, always an uncertain source of income and not to be relied on. Some hospitals carried their legacies to a reserve fund, but they had always been obliged to use their's to pay their debts. Their expenditure for the past year amounted to £14,093, which was £150 less than in 1904. Their receipts were, therefore, £600 less than their expenditure, which was a very unsatisfactory state of affairs. The hospital had never as yet been in debt, but it was only the payment of £1,000 from the Zunz fund which had enabled them to pay the tradesmen's accounts for the Christmas quarter.

Mr. C. W. Blackman, Chairman of the Weekly Board, seconded the resolution and remarked that the nursing department had undergone a great change since the arrival of their new matron and much reorganisation had taken place. One great change was the appointment of wardmaids to do the rougher work in place of the probationers, who would thus be enabled to devote more time to instruction, etc. This change entailed greater expense and he appealed to the Governors to try and get fresh subscribers to the hospital. The Ladies' Association had been doing very good work, and were promoting an entertainment to be held at His Majesty's Theatre, by kind permission of Mr. Tree, on May 22. He trusted all present would attend and help to make it a success. The hospital had derived much benefit from the Dresden Assistance Fund, which had enabled them to send many of their in-patients to a convalescent home, and thereby ensure their complete recovery before returning home.

A vote of thanks to Princess Louise Augusta of Schleswig-Holstein and the ladies of the Association for their valuable help to the hospital was proposed by the chairman, who said that one of the advantages of the Royal Free Hospital was that the work done there was not confined to the male element. They were very grateful for the most important part played by the ladies. They owed much to the enormous interest that Princess Christian and her daughter always took in the hospital. They regretted the loss of Miss Wedgwood, their late matron. The new matron, Miss Cox Davies, seemed to be admirably fitted for her post.

The vote was seconded by Mr. Montagu Ellis.

A vote of thanks to the medical staff was proposed by Major Ricardo and seconded by Sir Edmund Hay Currie, who said that as Secretary of the Hospital Sunday Fund he had much pleasure in bearing testimony to the splendid reputation of the hospital and the high professional position of the staff. With regard to the Ladies' Association connected with the hospital, he had recently had a great deal to do looking into the question of the abuse of out-patients. There was no great abuse, the name was a wrong one, but if it was to be checked at all it must be by means of almoners. There was no hospital in London where the almoners did a greater work than was done at the Royal Free by two excellent ladies, thoroughly well trained and well qualified. The Royal Free Hospital was setting a grand example by employing these two lady almoners, and supplementing their work by the help of other ladies.

The proceedings terminated with a vote of thanks to the chairman.

### TOTTENHAM HOSPITAL.

It is very evident from the speeches which follow that a wider interest in the work of this hospital must be aroused in the district if the finances are ever to assume a prosperous condition. The Committee deserve well of the community they serve, for they have shown much public spirit by bringing the hospital up to modern requirements. A Hospital League should be formed by the ladies, with captains of streets, so that each street may have its penny collection, and in this way the additional income of £3,000 a year should be forthcoming. What the Tottenham Hospital evidently needs is the help of a few earnest people who recognise the privilege of giving a measure of personal service in the days of health in the cause of the sick. This the late Mrs. J. P. Beavan did, and her loss, by depriving the Ladies' Association of an able honorary secretary with initiative, has been eloquently shown by the drop in the contributions from this source. Mrs. Beavan's services should be suitably commemorated in connection with this hospital, and we cannot but believe that others will be found to take her place. No hospital is more needed than the Tottenham Hospital, and few have greater claims to the gifts of the more intelligent of hospital supporters in the Metropolis.

Sir Francis Cory-Wright, Chairman of the Board of Governors, presided on the 27th ultimo at the annual general meeting of the Tottenham Hospital. There was a good attendance of subscribers and donors.

The Chairman moved the adoption of the report and accounts. It would be seen, he said, that their position was £1,986 worse than last year, for whereas they then had a debit balance of £1,735, they now had a deficiency of £3,721. That, however, was easily explained. This year there was no Tottenham Carnival, and last year that brought them in £857. This year the Ladies' Association had raised £659 less, but they could not expect them to get up a bazaar every year. Last year they had rather more donations, and they took £800 from the dinner; the difference together making a decline of £1,005. The penny collections, too, he was sorry to say, had yielded £57 less. Their annual subscriptions were £207 more, the Sunday Fund gave them £257, and the Saturday Fund £17 more; while their "other receipts" were up by £79, and they had a legacy of £100. Then on the other side of the balance-sheet they had had 176 more in-patients and 929 more out-patients and casualties; but the increased expenditure of only £357 showed that if they worked the hospital up to the hilt they brought the standing expenses down to the limit. All through the year they had averaged 72 beds occupied. In 1903 the average cost per head per in-patient was £4 6s. 4d.; in 1904 they got it down to £3 17s. 3d., and in 1905 to £3 10s. 8d., and had received from King Edward's Fund special praise for economical management. Last year King Edward's Fund gave them £3,000, earmarking £1,000 for buildings; this year out of the same amount they earmarked £2,000 for buildings. They had been engaged for some time in extending the hospital, and this had been absolutely necessary, as the district grew in population. They had made more room, and it would be the fault of those who failed to find the necessary funds if they did not take in more patients. With their 58 extra beds they could now accommodate 800 more patients a year, but they would require another £3,000 per annum to do it with. If the money were not forthcoming the wards would not be opened.

Mr. J. Howard seconded the resolution, which was carried, as was also the re-election of the seven retiring members of the Board, with Mr. Horace Bryant to fill a vacancy. Votes of thanks brought the proceedings to a close.

### KING'S COLLEGE HOSPITAL.

The chair was taken at the annual meeting of this hospital by the Rev. Dr. Headlam, Principal of King's College.

In moving the adoption of the annual report and balance-sheet, the Chairman said that they had a very difficult task on hand at the present time—namely, to keep the hospital going, and also to collect sums of money towards the removal fund. Owing to exceptional good fortune, they had been able to pay their way during the past year, but this was due to an unusual sum in the way of legacies; their ordinary annual income had not increased. Unless there was some increase in the present year they would be unable to pay their way. The rates of the hospital had been considerably increased, which would be an additional expense.

The Committee had been unfortunately compelled to accept the resignation of Miss Monk on account of ill-health, who had held the office of Sister-Matron for 21 years, and had served the hospital faithfully, giving to it more strength than she could spare. The Committee highly appreciated her invaluable and devoted services, and felt that her loss, coming as it did when the hospital was involved in the difficulty of removal, was a great blow.

The adoption of the report having been seconded, Sir Charles Ollivant said that he had been asked by Lady Methuen, wife of their Chairman, and other ladies, to help them in organising a fête with the object of raising funds for the removal of the hospital. He appealed to all present to help in whatever way they could. It was most desirable that the neighbourhood into which the hospital was to be moved should be stirred up to join in the work of collecting funds. He hoped they would have a meeting in Camberwell to which the people of that part should be urged to come, and afterwards branch meetings in the various surrounding districts.

Before the conclusion of the meeting a question was put to the Chairman with regard to the progress of the fund for the removal of the hospital.

The Rev. Dr. Headlam, in reply, said that they had begun by issuing an appeal for £300,000, to include the cost of building up to a certain point and the site. Towards that they had received just half the amount, including value of site, and they had now full possession of that site. For the purpose of building they had a substantial sum in hand, which would enable them to build at once. During the past year they had had a large amount of business to do in connection with selecting an architect and preparing working drawings. Mr. W. A. Pint had been selected as architect, and the drawings which he had prepared had met with the highest approval of all interested in the matter and of all hospital experts. They had instructed Mr. Pint to prepare working drawings for the out-patients' department. This would take two months; as soon as the drawings had been prepared and approved they would get out contracts, and hoped to be able to begin to build the out-patients' department in the course of the next three months. They had sufficient money in hand to see them to the end of the year. They must impress upon the public the fact that they meant to get on with the building of the hospital as rapidly as they could. They had not yet got sufficient money to enable them to finish building, and must continue their appeal. The fête mentioned by Sir Charles Ollivant would take place in the month of May in Lincoln's Inn Fields, by permission of the Benchers. He hoped that a large number of persons would help them in this work. It had been thought better not to press the appeal for some time, but they were now taking it in hand energetically. They hoped to be able to go on building continuously. They had received another donation of £4,000 from King Edward's Hospital Fund towards the removal, and various other donations.

### MIDDLESEX HOSPITAL.

A good number of Governors assembled at the Middlesex Hospital on the occasion of the annual meeting on February 22.

The chair was taken by H.S.H. Prince Francis of Teck.

In the unavoidable absence of Lord Cheylesmore, chairman of the Weekly Board, the adoption of the report and balance-sheet was moved by Colonel Needham, who said that during the past year 3,147 in-patients and 36,817 out-patients had received attention, which, taking into consideration the fact that the hospital had been closed for eight weeks in the summer on account of repairs, showed an increase in all departments. The cost per bed was £96 6s. 6d., and the cost of each in-patient per week was £2 8s. 0½d.

The ordinary income showed a decrease of £1,035. This difference was accounted for under the heading of annual subscriptions, the reduction in the amount of which was attributable to the discretionary powers exercised under the special authority and direction of the subscribers. The donations were much the same as in 1904, the amount being £1,946. There was a great depreciation of dividends in consequence of the unavoidable sale of stock, the receipts under this heading being less by £603 than in 1904. This had, however, been counterbalanced by the very generous grant from the Hospital Sunday Fund of £3,235, while £1,000 had again been received from King Edward's Hospital Fund, and £500 from the Hospital Saturday Fund. They had received £3,156 more through legacies in the past year, including one of £9,022. The decrease in ordinary expenditure was mainly due to the closing of the wards. The subject of expenditure had been receiving most careful attention, and a special committee had been appointed to investigate the matter. The extensive and urgent repairs to the hospital had all been carried out satisfactorily. One important addition had been the providing for a complete sterilising plant for the operating theatres, and it was gratifying to trust that in that department the hospital would bear favourable comparison with the best in the Kingdom. Mr. Bischoffscheim had generously defrayed the cost of lining a second ward with "Crystopal," and they trusted the other wards would be similarly treated at an early date. After referring in terms of great regret to the resignation of Mr. Henry Morris he went on to say that Miss Thorold had also severed her connection with the hospital after thirty-five years' work as lady-superintendent. Her place had been filled by Miss Vernet, to whom they looked with every confidence to uphold the high traditions of the past.

As regards the financial position of the medical school, it would be remembered that the executive committee of King Edward's Hospital had notified that in future no grants would be made from that fund to any hospital which made payment to its medical school out of general funds. At a special meeting it was decided to establish a permanent endowment fund for the maintenance of the school, towards which Mr. Henry Morris gave £1,000.

It could not be denied that the financial position of the hospital was very serious and they were faced with apparently insurmountable difficulty as to how to obtain money for the maintenance of the hospital in an efficient condition. Nowhere was the strenuous trend of life so felt as in hospitals, where, as well as the increase in population and patients, they were faced with the expensive demands of modern science; while, on the other hand, contributions tended to decrease. He could only trust in the well-known generosity of the public to help them out of their difficulties.

The resolution was seconded by Mr. Herbert Ormerod, K.C., who, in advocating the establishment of a permanent endowment fund for the medical school, spoke very strongly of the benefit to the hospitals and the world at large of the maintenance of these schools for the training of medical students.

The other business being disposed of, Prince Francis of Teck, in reply to a vote of thanks, said he hoped some good would come out of the difficulty in which the hospital found itself as regards funds, and trusted they would all do what they could to obtain funds. It was very unfortunate that during the past year they had had to spend £12,000 in making the old building habitable, and also that all the work had to be done at once. He had noticed that the people who when in the country supported their local hospital and nursing fund, when they came to London never seemed to feel the responsibilities that they owed to the great London hospitals. Year in, year out there were a large number of people living in London who never subscribed to any hospital. He trusted they would all try and get their friends to subscribe any sum, however small, so that each hospital could be supported in its own sphere of influence. Everyone should feel as responsible for the hospitals as for the payment of rates. He did not want to see the hospitals paid out of the rates, and to prevent this there must be a large increase of annual subscribers.

### CHARING CROSS HOSPITAL.

The annual meeting of Governors of this hospital was held on February 21, the Hon W. F. D. Smith, M.P., in the chair.

The Chairman, in moving the adoption of the report, said that the new surgical ward had been opened during the past year, and 90 more beds had thereby been added to the accommodation of the hospital, but as 51 beds had been put out of use they had now a total of 187. All who had seen the new ward had remarked on its excellent arrangements and general efficiency for its purpose. They had also a new operating and lecture theatre. He wished it was possible to congratulate them upon the satisfactory condition of the finances of the hospital. He would have liked to be able to tell them that the new buildings had been paid for, but, unfortunately, there was now, in addition to the mortgage for £85,000, a debt of £6,400 owing on the building and furnishing account, which it was the object of the hospital to pay off as soon as possible. There was also a very considerable deficit in the annual working of the hospital, which could only be met by temporary and special appeals, or by a considerable increase in the number of annual subscribers. In face of these conditions they had decided to postpone the completion of the second part of the renovation of the hospital until the present work had been paid for. During the present year a special appeal would have to be made for funds to clear off the debt on the building and the annual deficit. A meeting would be held at His Majesty's Theatre, by permission of Mr. Tree, on March 15. Three gentlemen of the Board had promised to give £200 each towards a fund to be called "The Emergency Fund," and they trusted that before the meeting in March two more gentlemen would promise £200 each, so as to bring the total up to £1,000. He much regretted the retirement of Mr. George Drummond who had done such invaluable work for the hospital as Treasurer. They were fortunate in having secured Mr. Malcolm to fill that post. If there had been any need for justification of the position of the hospital, they had only to point to the work they had been able to do at the time of the Charing Cross disaster. It was a fact that within a very few moments the staff of the hospital were on the scene of the disaster, and within an hour 33 cases had been treated within its walls.

The resolution having been seconded by General Sir Alexander Badcock, the customary votes of thanks were passed.

### BROMPTON HOSPITAL FOR CONSUMPTION.

The proceedings at the quarterly court of governors were of a purely formal character. The chair was taken by Lord Cheylesmore, and the report of the Committee of Management was submitted and approved without any speeches being made.

The report shows that Princess Victoria of Schleswig-Holstein paid a visit to the sanatorium and convalescent home on December 19, when a Ladies' Guild was formed.

In conjunction with the medical staff the Committee of Management have formed a sub-committee to consider by what means they can increase the utility of the hospital and prevent the increase of the disease, which they hope may prove of great benefit to the public. The Royal Sanitary Institute has expressed much interest in the proposal, and at the invitation of the Committee has appointed Dr. Newsholme, Medical Officer of Health for Brighton, as their representative to assist the sub-committee.

A grant of £1,000 has been received from King Edward's Hospital Fund, £425 from the Hospital Saturday Fund, besides a good number of other donations.

The Medical Committee's report shows that very satisfactory results are experienced by patients treated at the sanatorium and convalescent home, but there is a most urgent need for funds towards its maintenance.

During the last quarter 414 patients have been received; 379 discharged, many greatly benefited; 74 transferred to the sanatorium and convalescent home; and 36 died. There have been 1,954 new out-patient cases.

### QUEEN CHARLOTTE'S LYING-IN HOSPITAL.

The annual court of governors was held on February 26, when the chair was taken by Sir Samuel Scott, M.P.

The Chairman, in moving the adoption of the report, said that during the year there had been a considerable increase in the number of both in-patients and out-patients. The number of in-patients was 1,560, an increase of 104. The daily average number of patients was 57, and the average stay 13.5 days. Five patients died: in each case the patient when admitted was in the gravest possible condition. The total of out-patients was 1,969, being 215 more than in 1904. As there had been a large increase in the number of patients there had necessarily been also a corresponding increase in the ordinary expenditure of £338. He was sorry to say that there was also a decrease in the ordinary income of £206, and the income was therefore £1,281

less than the ordinary expenditure. They were especially sorry as the expenses had gone up.

The friends and relations of the late Earl of Hardwicke had contributed £1,000 in order to found a bed to his memory.

The new fund started by Mr. Nesden for the purpose of assisting in-patients of the hospital had proved most beneficial. There had been an increase in the patients admitted into the Convalescent Home, and the Committee had been considering whether it might not be possible to increase the accommodation at the home, but this was a matter for future consideration. One of the most important adjuncts of the hospital was the Nurses' Home. It had been found that the accommodation there was insufficient to meet the needs. A site had been acquired, but a large sum of money was necessary to carry out the building of the proposed increase to the Nurses' Home. A special appeal had been made for £10,000, but up to the present only £1,556 had been received. They must appeal very earnestly to the public for further contributions towards this building. No one benefited more than did the public by the training given to the nurses of this hospital in that home. He trusted they would all make great efforts during the present year to obtain funds to meet the increased expenditure, caused by the greater number of patients.

The resolution was seconded by Mr. F. W. Hunt, and after various other resolutions had been passed, the proceedings terminated.

### ADDENBROOKE'S HOSPITAL, CAMBRIDGE.

In our issue of February 17 we published an appeal which Mr. Kett and Miss Caroline Stephen have issued for the building fund for the Addenbrooke Convalescent Home. A wish is now expressed that we shall make it clear that the Convalescent Home, though intended for the exclusive use of Addenbrooke's Hospital, is quite a separate institution with separate funds, and its own committee. It is further desired that it shall be stated that the appeal was made by the committee of the Home, and not by that of the hospital.

*** We regret that, owing to pressure on our space, the reports of several hospital meetings have been unavoidably held over till next week.

Miss Lawson, of Arkleby Hall, Aspatria (a sister of Sir Wilfrid Lawson, M.P.), bequeathed in her will legacies of £1,000 to the London Temperance Hospital, and £1,000 to the Cumberland Infirmary, Carlisle.

